# Social media users’ attitudes toward cyberbullying during the COVID-19 pandemic: associations with gender and verification status

**DOI:** 10.3389/fpsyg.2024.1395668

**Published:** 2024-06-13

**Authors:** Lifang Li, Jiandong Zhou, Sally McManus, Robert Stewart, Angus Roberts

**Affiliations:** ^1^School of Journalism and Communication, Sun Yat-sen University, Guangzhou, China; ^2^Division of Health Science, Warwick Medical School, University of Warwick, Coventry, United Kingdom; ^3^Violence and Society Centre, City, University of London, Northampton Square, London, United Kingdom; ^4^Department of Psychological Medicine, King’s College London, London, United Kingdom; ^5^South London and Maudsley NHS Foundation Trust, London, United Kingdom; ^6^Department of Biostatistics and Health Informatics, King’s College London, London, United Kingdom

**Keywords:** cyberbullying, COVID, gender, verification status, emotional responses

## Abstract

**Introduction:**

Social media platforms such as Twitter and Weibo facilitate both positive and negative communication, including cyberbullying. Empirical evidence has revealed that cyberbullying increases when public crises occur, that such behavior is gendered, and that social media user account verification may deter it. However, the association of gender and verification status with cyberbullying is underexplored. This study aims to address this gap by examining how Weibo users’ gender, verification status, and expression of affect and anger in posts influence cyberbullying attitudes. Specifically, it investigates how these factors differ between posts pro- and anti-cyberbullying of COVID-19 cases during the pandemic.

**Methods:**

This study utilized social role theory, the Barlett and Gentile Cyberbullying Model, and general strain theory as theoretical frameworks. We applied text classification techniques to identify pro-cyberbullying and anti-cyberbullying posts on Weibo. Subsequently, we used a standardized mean difference method to compare the emotional content of these posts. Our analysis focused on the prevalence of affective and anger-related expressions, particularly examining variations across gender and verification status of the users.

**Results:**

Our text classification identified distinct pro-cyberbullying and anti-cyberbullying posts. The standardized mean difference analysis revealed that pro-cyberbullying posts contained significantly more emotional content compared to anti-cyberbullying posts. Further, within the pro-cyberbullying category, posts by verified female users exhibited a higher frequency of anger-related words than those by other users.

**Discussion:**

The findings from this study can enhance researchers’ algorithms for identifying cyberbullying attitudes, refine the characterization of cyberbullying behavior using real-world social media data through the integration of the mentioned theories, and help government bodies improve their cyberbullying monitoring especially in the context of public health crises.

## 1 Introduction

Social media enables positive and negative communication, including cyberbullying (Jatmiko et al., 2020). Cyberbullying is defined as the use of the internet to send harassing or threatening messages, or post humiliating comments (Hinduja and Patchin, 2010). The effects of cyberbullying are potentially more severe than those of physical or verbal bullying because wider audiences can be reached and materials may be accessed repeatedly (Li and Peng, 2022), resulting in victims potentially reliving denigrating experiences (Hinduja and Patchin, 2010). As online access grows, the number of people exposed to cyberbullying may also increase (Beran and Qing, 2005).

Cyberbullying perpetration is associated with problematic internet use, defined as the psychological, social, school or work difficulties experienced because of using the internet (Yudes et al., 2021). During the COVID-19 pandemic, quarantine led many people to rely more heavily on text messages and social media, with an increased risk of cyberbullying (Cheng et al., 2020; Babvey et al., 2021; Morales-Arjona et al., 2022; Yang et al., 2022). In China and Malaysia, patients with COVID-19, especially those ‘super-spreaders’ who were confirmed as positive in certain cities and exposed by the media, were aggressively cyberbullied (Patel, 2021; Ting and Shamsul, 2022). Victims of cyberbullying often experience mental health harms as a result, including depression (Yudes et al., 2021) and suicide (Hinduja and Patchin, 2010; John et al., 2018). Researchers suggest that it is necessary to research cyberbullying behavior extensively (Bansal et al., 2023).

Investigations into the factors associated with social media users’ cyberbullying behavior could therefore benefit research and policy-making in this area: for example, efficient invention policy design and implementation can help reduce and mitigate the negative impact of cyberbullying. (1) Self-control theory has been utilized to argue potential gender differences in engaging in cyberbullying behavior, suggesting that females are less inclined to participate in cyberbullying compared to males (Griezel et al., 2012; Wong et al., 2018; Marr and Duell, 2021); (2) anonymity show positive influence in engaging cyberbullying behavior based on Barlett and Gentile Cyberbullying Model (BGCM) (Barlett et al., 2021a); and (3) individuals with low affective empathy demonstrated higher cyberbullying scores (Ang and Goh, 2010). More specifically, following General Strain Theory (GST), anger was found to be an important mediating factor of cybervictimization and cyberbullying (Ak et al., 2015).

However, few studies have integrated these factors (gender, anonymity, and affective) to find out their effects on cyberbullying attitudes using real-world social media data. Analyzing the interplay between gender, anonymity, and emotional factors is key to gain a comprehensive understanding of cyberbullying behaviors. There also lacks studies employing actual social media data for this purpose.

We aimed to fill these research gaps using the cyberbullying experiences of the COVID-19 patents during the COVID-19 pandemic, as previous study revealed a positive correlation between proximal experiences with COVID-19 and cyberbullying (Barlett et al., 2021b). Specifically, we would like to test social media users’ attitudes of pro-and anti-cyberbullying towards the patients. This interest arises from the observation that COVID-19 patients, particularly those whose travel routes have been disclosed, have been targets of cyberbullying (Lian et al., 2022). Using social media (Weibo) data created during the COVID-19 pandemic, this study aimed to:

1. Investigate the relationship between gender and the likelihood of sharing pro-cyberbullying content on Weibo.
2. Explore the connections between the verification status, gender, and cyberbullying attitudes of Weibo users.
3. Analyze how verification status, gender, and the use of emotional words are associated with the number of reposts (retweets) for pro-cyberbullying and anti-cyberbullying content.

To achieve the research objectives mentioned above, this paper introduced relevant theories in Section 2, discussed research methods in Section 3, presented research results in Section 4, summarized research findings, corresponding theoretical and practical implications, and limitations in Section 5, and finally concluded the paper in the last section.

## 2 Theoretical background

### 2.1 Gender and cyberbullying

Self-control theory refers to the capacity to delay immediate gratification, manage negative emotions, sustain perseverance in fulfilling obligations, and restrain impulsive behaviors (Kochanska et al., 1996). It entails regulating emotions, beliefs, and actions to cultivate healthy interpersonal relationships. According to this theory, self-control differs based on individuals’ gender, with males typically exhibiting lower levels (Wu et al., 2023). Historically, females have been socialized to conform to societal norms, promoting self-regulation and risk aversion, thereby reducing the likelihood of engaging in criminal behaviors (Wong et al., 2018). Self-control theory was applied to explain cyberbullying behavior (Stults and You, 2022). Consequently, researchers aimed to investigate how gender influences cyberbullying (Wang et al., 2019; Marr and Duell, 2021). However, findings in this regard are inconsistent.

One survey-based study found no significant difference of gender in cyberbullying behavior, with respect to COVID-19 experiences (Barlett et al., 2021c). Another group of studies have found males to be more likely to engage in cyberbullying (Barlett et al., 2021c), and other studies have found women to be more likely (Görzig and Ólafsson, 2013). Gender differences in cyberbullying victimization and suicidal ideation has also been found, with the association being stronger in women (Machimbarrena et al., 2018). However, there has been limited understanding of how gender influences the attitudes of males and females towards cyberbullying, despite attitudes being a crucial factor in predicting future cyberbullying behavior according to BCGM (Barlett, 2017). In this way, this study aimed to investigate whether females are less inclined than males to share pro-cyberbullying posts.

### 2.2 BGCM and social media users’ verification status

The BGCM posits that perceptions of anonymity and the belief in the irrelevance of physical stature are two interconnected cognitive structures that forecast cyberbullying attitudes (Barlett and Gentile, 2012). It also suggests that having positive attitudes toward cyberbullying predicts future engagement in cyberbullying behaviors (Barlett et al., 2017).

In social media platform, verified users, i.e., those who have uploaded proof of their identity to the platform, hence may be perceived less anonymity according to BGCM. Perceived anonymity shows positive effects on positive attitudes towards cyberbullying, which in turn has positive effects on future cyberbullying perpetration (Barlett and Gentile, 2012). Hence, unverified users, who may be perceived more anonymity than verified users, they may be more likely to share pro-cyberbullying posts. We will test it using our dataset.

In addition, been verified is helpful for users to be more central to the information retweeting network (González-Bailón and De Domenico, 2021). For instance, verified users on Twitter, under its previous management, have been identified by Twitter as accounts of public interest (Simon et al., 2014). Public figures are likely to be verified users (Wang and Zhu, 2021). Also, verified users tend to be more active, as was illustrated in the major spike in Twitter use from verified users when China experienced its first COVID-19-related death (Chen et al., 2020). This is anticipated because public figures and news sources frequently report breaking news immediately, and verified users play a prominent role in disseminating information as messages from verified media accounts are commonly shared through retweets. (González-Bailón and De Domenico, 2021). On Weibo, only around 17% of users are verified. If a verified user shares inappropriate information, they will be suspended by the platform for a period (Zhang and Lu, 2016). For famous verified users such as celebrities, sharing inappropriate information may harm their reputation and public image (Wang et al., 2014); Therefore, this also led to the hypothesis that verified users on Weibo exercise greater caution in their online behavior regarding pro-cyberbullying content compared to unverified users.

Existing research and theories suggest a negative association between verification status and pro-cyberbullying attitudes. However, despite this association, verified users’ higher activity and influence on social networks amplify the reach of their pro-cyberbullying content. The expedited dissemination of posts from verified users may be attributed to the preference for information from trusted sources, which demands less cognitive effort compared to content that contradicts established beliefs or perspectives (Knobloch-Westerwick et al., 2020). Moreover, as individuals share or repost content that could be perceived as cyberbullying, it may contribute to perpetuating such behaviors in an amplified manner among online users (Steinmetz et al., 2014). Therefore, we will examine whether or not pro-cyberbullying content from verified users on Weibo is more likely to be reposted than that from unverified users.

### 2.3 Emotions and cyberbullying

The increased risk of cyberbullying during the pandemic may be because people feared exposure to the virus, because of reduced income, fear of death or hospitalization, or stigmatization (Yang et al., 2022), all stressors that are related to emotional problems such as increased use of emotional expressions, depression, or anxiety (Holmes et al., 2020; Wong et al., 2020; Barlett et al., 2021b). Researchers established the framework for a General Strain Theory (GST) with a core emphasis on negative emotions and affect (Agnew and White, 1985). This theory posits that negative affective states, such as anger and related emotions, emerge in response to certain stimuli, thereby heightening the likelihood of delinquent adaptations (Mazerolle et al., 2000). GST has been extensively utilized to analyze aggressive behaviors, including cyberbullying (Lianos and McGrath, 2018). Using a sample of 1,103 Chinese adolescents survey, researchers found that adolescents facing financial strain are at a heightened risk of experiencing emotional challenges such as anger and depression, which in turn, increase the likelihood of engaging in cyberbullying perpetration (Wang and Jiang, 2023).

In addition, previous studies have found that emotions are closely related to the occurrence of cyberbullying and that the expression of emotions has gender characteristics (Li, 2006; Armenti and Babcock, 2021; Santos et al., 2021). For example, women were found to use more emotional words in comparison with men when defending their image, especially in relation to anger (Paciello et al., 2020). Anger expression is consistent with the external attribution of blame (Rico et al., 2017), which also suggests a relationship between cyberbullying and expressions of anger. Experiencing anger is linked to higher participation in cyberbullying (Wollebæk et al., 2019), possibly because individuals experiencing anger tend to respond aggressively and engage in cyberbullying more frequently (Den Hamer and Konijn, 2016). Gender-specific differences were also found in experiences of online sexuality and intimacy, and aggressive and problematic online encounters (Chang et al., 2021). In contrast to males, females, who typically possess higher levels of affective and cognitive empathy, are less inclined to engage in online aggression (Ang and Goh, 2010). Therefore, we aim to test whether in females’ pro-cyberbullying posts there are less anger/affect words than in males’ pro-cyberbullying posts.

Given that anonymity (verified status) and gender are recognized as influential factors in cyberbullying behavior, with affect and anger serving as potential mediators of cyberbullying attitudes, our study is also interested to examine the variations in affect and anger within pro-cyberbullying posts among verified and unverified individuals of both genders.

## 3 Materials and methods

This section introduces the social media data collection, preprocessing, processing (annotation and classification), emotion words extraction, statistical analysis, and robustness check.

### 3.1 Data collection and preprocessing

Before collecting data, we need to confirm the searching keyword that can be applied. Our goal was to analyze cyberbullying-related post related to people diagnosed with COVID-19. We used Baidu Index and Weibo search results to identify keywords and phrases. Baidu Index,^1^ similar to Google Trends,^2^ has been used for keyword selection and filtering (Vaughan and Chen, 2015; Fang et al., 2021). It measures the popularity and relevance of search terms in online discourse, providing a quantitative measure of their significance. Both keyword stemming and related keyword generation methods can facilitate the identification and retrieval of documents relevant to a specified keyword. Researchers used Baidu Index and identified keywords that could accurately represent terms commonly linked with influenza epidemics (Yuan et al., 2013). We will employ the Baidu Index to identify cyberbullying-related keywords associated with COVID-19.

**Figure 1:**
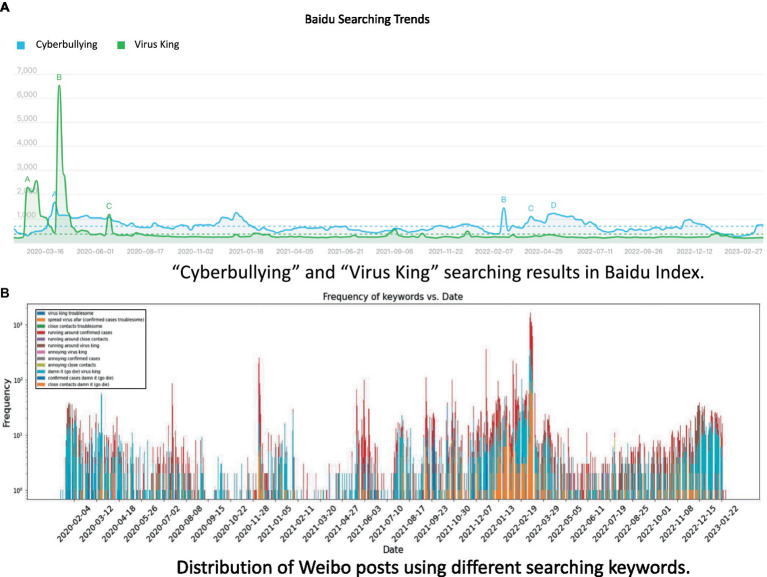
Baidu Index searching results and distribution of posts’ searching results. **(A)** “Cyberbullying” and “Virus King” searching results in Baidu Index. **(B)** Distribution of Weibo posts using different searching keywords.

Firstly, we searched for “cyberbullying” and “virus king” (This search term could be translated into English as ‘poison king’ or ‘virus king’, see the searching results in Figure 1A) in Baidu Index to gain initial insights on the range of relevant terms. This is because cyberbullying is the topic we focus on and “virus king” is a term used to refer to the stigma experienced by super-spreaders of coronavirus during COVID-19 pandemic (Ting and Shamsul, 2022).

Secondly, we manually checked the top 10 monthly search results for “cyberbullying” and “virus king” on Weibo from January 1, 2020, to February 28, 2023. This step involved identifying relevant keywords with similar meanings to “cyberbullying” and “virus king” from the top 10 search results. We examined 260 posts and a panel consisting of authors 1 and 2 identified two keywords per page, specifically focusing on keywords associated with resentment (Inner Annotator Agreement of F measure = 0.91).

From this analysis, we prepared the most frequent related keywords that can be applied for collecting Weibo posts. These keywords could be translated into English as: “virus king troublesome,” “spread virus afar (confirmed cases troublesome),” “close contacts troublesome,” “running around confirmed cases,” “running around close contacts,” “running around virus king,” “annoying virus king,” “annoying confirmed cases,” “annoying close contacts,” and “damn it (go die) virus king,” “confirmed cases damn it (go die),” or “close contacts damn it (go die)”.^3^

Data were collected from the Weibo API^4^ using keyword searching approach. We obtained in total 33,484 unique posts with at least one of the 12 keywords/phrases. For each post, the poster’s username, gender, verification status, the content of the post, and the time of the post were recorded. The visualization of the availability of these 12 keywords with dates was illustrated in Figure 1B for clarification.

### 3.2 Data processing

We conducted two data labeling steps to define the classification tasks of interests.

(1) Identify whether the posts were cyberbullying-related or not. Cyberbullying-related or not indicate that the post is about the cyberbullying topic or not, if the ‘cyberbullying-related or not’ was labeled as ‘yes’. Specifically, cyberbullying-related posts were defined as those which mentioned one or more of the 12 keywords and which annotators felt constituted cyberbullying directed towards COVID-19 patients. For example, “During these past few days, I got into arguments with several trolls on Weibo due to the Putian COVID-19 outbreak. Cyberbullying is extremely terrifying during a public health crisis. Nobody wants to get infected with the coronavirus, and patients who strictly follow the quarantine regulations are also innocent.” This post was labeled as “True” for being cyberbullying-related. However, it was labeled “False” for the post “Shanghai has reported 4 new locally transmitted confirmed cases. We remind everyone that the epidemic is still ongoing, so please do not let your guard down. Wear masks, avoid wandering aimlessly, and refrain from unnecessary travel.” In this step, we labeled 1,500 randomly selected posts for whether they were cyberbullying-related. Among them, 565 (37.7%) posts were coded as cyberbullying-related posts and the remaining 935 (62.3%) as not cyberbullying-related. The average pairwise Cohen’s Kappa of the three coders was 0.72.

(2) Label the affirmed cyberbullying posts into three types: ‘pro-cyberbullying’, ‘anti-cyberbullying’, or unclear. Pro-cyberbullying posts were defined as posts/reposts with content that is harsh towards or unfairly critical of people with COVID, or those that directly called confirmed cases “virus king.” For example, “these confirmed cases are so unethical. They know they have COVID-19 but still wander around recklessly.” Or “Why do not those wandering confirmed COVID-19 cases just go die? They are so annoying.” Anti-cyberbullying posts/reposts with content that is critical of people/content classified as cyberbullying. For example, “Regardless, cyberbullying towards confirmed cases is wrong,” was labeled as “anti-cyberbullying.” Unclear posts were defined as posts where we could not tell the attitudes of the poster to cyberbullying. For example, “What is your opinion about the cyberbullying of confirmed cases?” was labeled as “unclear.” The average Cohen’s kappa value of three coders was 0.85 (0.85, 0.81, and 0.88 separately) suggesting good agreement (Viera and Garrett, 2005; Babvey et al., 2021).

Based on previous research we applied five classification methods to the manually labeled data: k-nearest neighbors (KNN), random forest (RF), Gradient Boosting Machine (GBM), Extreme Gradient Boosting (XGB), Multi-Layer Perceptron (MLP, a type of neural network), and decision tree (DT) (Kotsiantis et al., 2007). A brief review of the above-mentioned machine learning models can be found in Al-Garadi et al. (2019). For each method, the bag-of-words (tokenizing data using Python package ‘jieba’^5^) was applied to extract features and Term Frequency-Inverse Document Frequency (TF-IDF) was applied to assign weights to words. Performance evaluation metrics for model comparisons include positive predictive values (PPV, precision), negative predictive value (NPV), recall, and F1 score, and their confidence intervals (CI) obtained by using the bootstrapping approach (Cho et al., 1997). We then conducted two rounds of evaluations.

Firstly, we identified whether posts were cyberbullying-related or not, using 10-fold cross-validation over an internal validation dataset (randomly sampled from 90% of the labeled data). Performance comparisons were summarized in Table 1, where DT ranks the best model to predict cyberbullying posts. The hold-out performances of the DT model (using the same optimal parameters) predict the labels of the remaining 10% held-out data.

**Table 1:** Classifier performance for identifying whether posts are cyberbullying-related or not, using 10-fold cross-validation approach.

	Training 90% labeled data, CV = 10	10% blind data as test data			
	F1-score [95% CI]	Cyberbullying-related or not	F1-score [95% CI]	Precision [95% CI]	Recall [95% CI]
KNN classifier	0.80 (0.75, 0.85)	True	0.37 (0.15, 0.56)	0.45 (0.18, 0.73)	0.32 (0.12, 0.53)
		False	0.63 (0.48, 0.77)	0.58 (0.41, 0.74)	0.70 (0.52, 0.88)
Random forest	0.87 (0.83, 0.91)	True	0.30 (0.09, 0.51)	0.60 (0.22, 1.00)	0.21 (0.05, 0.39)
		False	0.71 (0.59, 0.83)	0.60 (0.44, 0.74)	0.89 (0.77, 1.00)
GBM	0.87 (0.83, 0.91)	True	0.41 (0.18, 0.63)	0.61 (0.30, 0.90)	0.32 (0.13, 0.56)
		False	0.71 (0.58, 0.83)	0.62 (0.47, 0.78)	0.84 (0.69, 0.96)
XGB	0.85 (0.79, 0.89)	True	0.47 (0.24, 0.67)	0.61 (0.33, 0.89)	0.39 (0.18, 0.61)
		False	0.71 (0.57, 0.83)	0.64 (0.47, 0.80)	0.81 (0.64, 0.96)
MLP	0.86 (0.82, 0.90)	True	0.24 (0.00, 0.46)	0.65 (0.00, 1.00)	0.15 (0.00, 0.32)
		False	0.72 (0.59, 0.83)	0.59 (0.44, 0.74)	0.94 (0.83, 1.00)
**Decision Tree**	**0.83 (0.78, 0.88)**	**True**	**0.51 (0.30, 0.71)**	**0.55 (0.31, 0.79)**	**0.49 (0.26, 0.71)**
		**False**	**0.66 (0.51, 0.80)**	**0.64 (0.46, 0.81)**	**0.70 (0.52, 0.87)**

Bold values mean the best performed model and its performances on training and test data.

**Figure 2:**
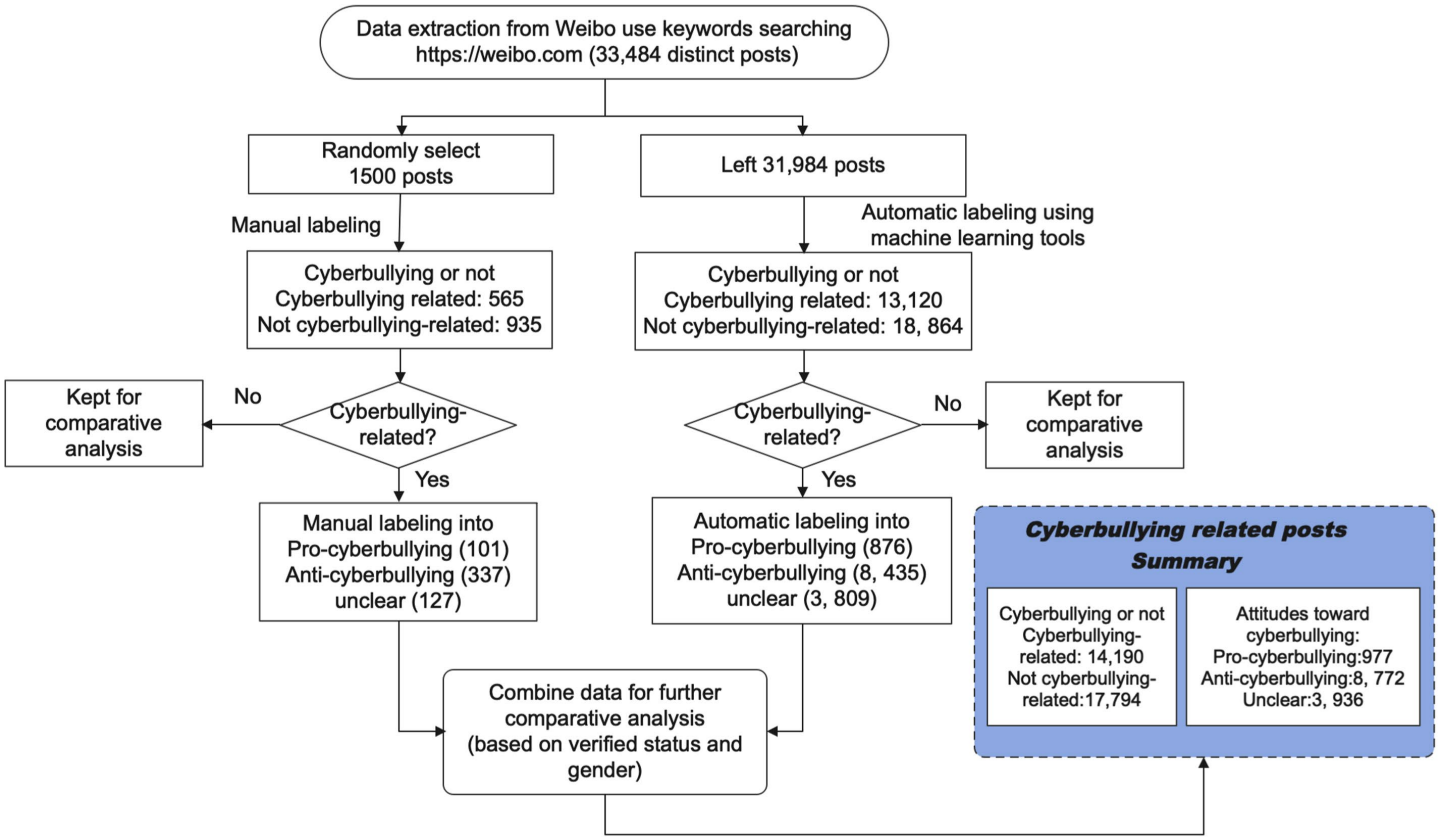
Data collection and pre-processing processes.

Secondly, we used these models to predict whether cyberbullying-related posts appear to be pro-cyberbullying or anti-cyberbullying (Table 2), and found that XGB achieved the best performance. And hold-out performances of XGB over the remaining 10% of labeled data. Regarding the performance of the best model (i.e., XGB) over the testing data, although binary prediction performance for the positive is not good, its prediction strength for the negative is good with (F1 = 0.79), indicating the classifier’s good performance in distinguishing negative from overall posts. This satisfying the choice the best prediction model for binary classifier, as has been used and interpreted by Yang et al. (2023). As seen in the obtained performance summary for predicting ‘cyberbullying-related or not’ (Table 2), the best model XGB achieved high performance for predicting prevalence-dependent negative instances (F1 = 0.79, with recall = 0.93), demonstrating its ability to exclude ‘cyberbullying’ from overall posts with high confidence. Figure 2 shows the number of each label.

**Table 2:** Classifier performances for whether cyberbullying-related posts appear to be pro-cyberbullying or anti-cyberbullying, using 5-fold cross-validation approach.

	Training 90% labeled data, CV = 5	10% blind data as test data, 90% data as training data			
	F1-score	Cyberbullying-related or not	F1-score [95% CI]	Precision [95% CI]	Recall [95% CI]
KNN classifier	0.75 (0.70, 0.79)	True	0.00 (0.00, 0.00)	0.00 (0.00, 0.00)	0.00 (0.00, 0.00)
		False	0.76 (0.66, 0.86)	0.65 (0.51, 0.79)	0.94 (0.85, 1.00)
Random forest	0.95 (0.93, 0.98)	True	0.02 (0.00, 0.17)	0.19 (0.00, 1.00)	0.01 (0.00, 0.09)
		False	0.80 (0.70, 0.89)	0.66 (0.53, 0.80)	1.00 (1.00, 1.00)
GBM	0.94 (0.91, 0.97)	True	0.16 (0.00, 0.42)	0.48 (0.00, 1.00)	0.10 (0.00, 0.29)
		False	0.78 (0.67, 0.88)	0.67 (0.53, 0.80)	0.95 (0.85, 1.00)
**XGB**	**0.92 (0.89, 0.95)**	**True**	**0.25 (0.00, 0.50)**	**0.56 (0.00, 1.00)**	**0.17 (0.00, 0.38)**
		**False**	**0.79 (0.68, 0.89)**	**0.69 (0.54, 0.82)**	**0.93 (0.83, 1.00)**
MLP	0.98 (0.96, 0.99)	True	0.16 (0.00, 0.42)	0.48 (0.00, 1.00)	0.10 (0.00, 0.29)
		False	0.78 (0.67, 0.88)	0.67 (0.53, 0.80)	0.95 (0.85, 1.00)
Decision Tree	0.90 (0.87, 0.94)	True	0.24 (0.00, 0.48)	0.42 (0.00, 0.83)	0.18 (0.00, 0.39)
		False	0.76 (0.64, 0.86)	0.67 (0.53, 0.82)	0.87 (0.74, 0.97)

Bold values mean the best performed model and its performances on training and test data.

### 3.3 Emotion words extraction from social media posts

To extract the number of emotion-related words, we applied the Linguistic Inquiry and Word Count (LIWC) tool (Tausczik and Pennebaker, 2010), which divides words into psychologically meaningful categories and has previously been found effective in extracting emotion words from Twitter posts and online reviews (Tumasjan et al., 2010; Del Pilar Salas-Zárate et al., 2014). We applied the LIWC simplified Chinese edition (2015) and conducted text segmentation using the Python “*jieba”* package to segment Chinese words (Zhang and Goncalves, 2016). We then summarized the rates of two categories of LIWC-based emotion words in the cyberbullying-related posts: affect (all kinds of emotions) and anger.

### 3.4 Statistical analysis

Descriptive statistics of the extracted characteristics from texts or other sources of data, continuous variables were presented as mean and standard deviation. The standardized mean difference (SMD) was applied to compare continuous variables’ differences in two targeted sets: posts pro-cyberbullying and anti-cyberbullying, within different groups of users based on their verification status and gender.

Standardized mean difference (SMD, also known as Cohen’s d measure) is given by the following Equation (1) for continuous variables (Hedges et al., 2012):

(1) $$SMD=\frac{\bar{X_{1}}-\bar{X_{2}}}{\sqrt[{2}]{\left(s_{1}^{2}+s_{2}^{2}\right)/2}}$$

where $X_{1}$ and $X_{2}$ are sample mean for the treated and control groups, respectively; $s_{1}^{2}$ and $s_{2}^{2}$ are sample variance for the treated and control groups. It is noted that the difference between two groups is no long dependent on the unit of measurement and thus variables with different types of measurements can be compared on SMD scale. The smaller the SMD, the smaller the difference in the corresponding covariates (Zhang et al., 2019). Although a threshold value such as 0.1 is not a fixed absolute standard because there is no mathematically accurate basis, a common rule of thumb for determining no group difference considering it to be achieved when the absolute value of SMD is less than 0.1 (Sun et al., 2023; Fadini et al., 2024). That is, if a SMD value is less than 0.1, the difference between the two groups is small. In our study, we used threshold value 0.2, which is even more strictly to define the difference between group comparison.

### 3.5 Robustness check

To ensure the persuasive validity of the examination results for posts targeting pro-cyberbullying, we manually annotated 977 posts that had already been predicted by machine learning models (one of our authors annotated the text, followed by a thorough double-check by another to ensure uniform understanding between them). The results showed that 904 posts were still labeled as pro-cyberbullying. This implies a machine labeling accuracy of 92.5%. Further statistical analysis (SMD) was conducted on the posts manually labeled as pro-cyberbullying, and the results have been included in Table 3 (row 3 and row 5) and Table 4 (row 3 and row 5). The same findings can be observed from the obtained analysis results, showcasing the robustness of the developed machine learning models for cyberbullying prediction from social media posts, and its potential to be used in larger scale for the identification and surveillance of cyberbullying during a public health crisis. Please find the labeled data using this link.^6^

**Table 3:** Summary of mean differences of emotion words frequencies from users of different gender and verified types in pro-cyberbullying and anti-cyberbullying posts.

	Emotional words frequency	Male verified Mean (SD); *N*; (1)	Male unverified Mean (SD); *N*; (2)	Female verified Mean (SD); *N*; (3)	Female unverified Mean (SD); *N*; (4)	SMD (1)–(2)	SMD (3)–(4)	SMD (1)–(3)	SMD (2)–(4)
Pro-cyberbullying	Affect	5.6 (9.3); *n* = 61	6.4 (7.6); *n* = 186	4.8 (7.6);*n* = 129	5.5 (7.4); *n* = 601	0.1	0.01	0.12	0.12
	Affect (labeled)	6.0 (9.5); *n* = 57	6.8 (7.6); *n* = 176	5.2 (7.8);*n* = 117	5.8 (7.6); *n* = 554	0.09	0.08	0.09	0.12
	Anger	0.8 (2.5); *n* = 61	1.8 (4.2); *n* = 186	1.4 (3.4); *n* = 129	1.4 (4.1); *n* = 601	0.29*	0.09	0.21*	0.09
	Anger (labeled)	0.8 (2.6); *n* = 57	1.8 (4.3); *n* = 176	1.5 (3.5); *n* = 117	1.5 (4.2); *n* = 554	0.29*	<0.01	0.22*	0.09
Anti-cyberbullying	Affect	6.6 (6.7); *n* = 891	8.4 (7.5); *n* = 1791	8.0 (7.6); *n* = 1,107	9.2 (8.0); *n* = 4,983	0.25*	0.15	0.19	0.11
	Anger	2.0 (3.0); *n* = 891	1.8 (3.1); *n* = 1791	2.0 (3.3); *n* = 1,107	2.0 (3.2); *n* = 4,983	0.07	0.01	<0.01	0.06

*Indicates SMD > 0.2.

**Table 4:** Summary of the posts from users of different gender and verification status.

		Male verified Mean (SD); (1)	Male unverified Mean (SD) (2)	Female verified Mean (SD) (3)	Female unverified Mean (SD) (4)	SMD (*p*-value) (1)–(2)	SMD (P-value) (3)–(4)	SMD (P-value) (1)–(3)	SMD (P-value) (2)–(4)
Pro-cyberbullying	Number of reposts	4.6 (12.5)	0.6 (2.8)	0.5 (2.6)	0.4 (4.9)	0.45*	0.03	0.48*	0.06
	Number of reposts (labeled)	4.8 (12.9)	0.6 (2.9)	0.5 (2.7)	0.2 (1.4)	0.45*	0.14	0.47*	0.20*
	Number of posts	61	186	129	601				
	Number of posts (labeled)	57	176	117	554				
Anti-cyberbullying	Number of reposts	134.0 (2280.1)	1.51 (23.14)	35.6 (356.2)	1.52 (22.71)	0.08	0.13	0.06	<0.01
	Number of posts	891	1791	1,107	4,983	/	/	/	/

*Indicates SMD > 0.2.

## 4 Results

### 4.1 Supportiveness of cyberbullying

**Figure 3:**
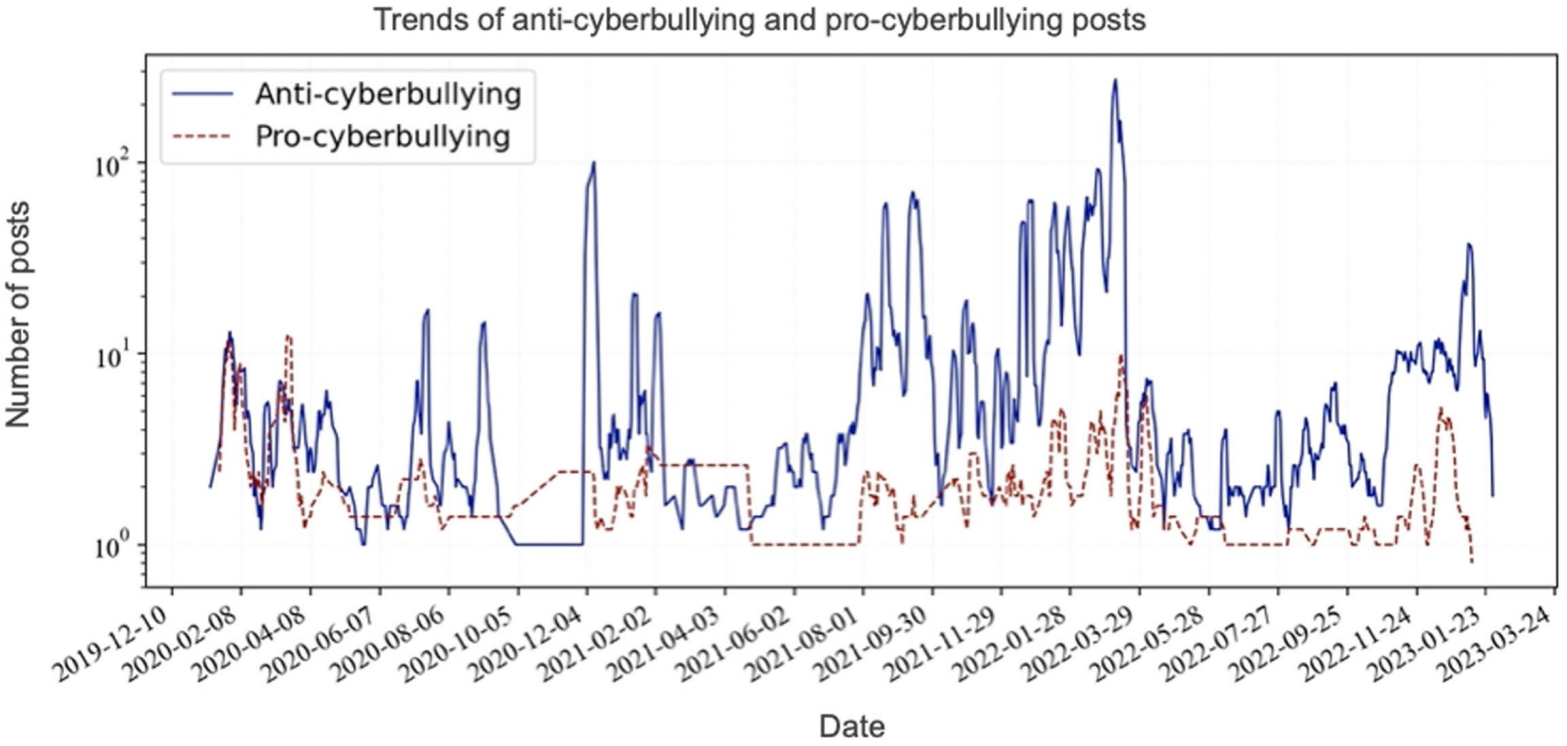
Temporal trends in the number of anti-cyberbullying and pro-cyberbullying posts, 2020 to 2023.

The daily distributions (smoothed) of posts anti-cyberbullying and pro-cyberbullying are presented in Figure 3.

Figure 3 shows that during the early months (from January 2020 to March 2020) after the start of the COVID-19 pandemic, the number of pro-cyberbullying posts was similar to the number of anti-cyberbullying posts. Later, anti-cyberbullying posts are much more frequent than pro-cyberbullying posts.

Further, the mean value and standard deviation (SD) were calculated for the number of affect/anger words in the pro-cyberbullying posts and anti-cyberbullying posts. Table 5 summarizes the differences between the pro-cyberbullying group and the anti-cyberbullying group.

**Table 5:** Comparison of pro-cyberbullying and anti-cyberbullying posts.

Characteristics	Pro-cyberbullying Mean (SD); (*N* = 977)	Anti-cyberbullying Mean (SD); (*N* = 8,772)	Standardized Mean Difference (SMD)
Affect (all kinds of emotional words, e.g., happy, sad, disgust etc.)	5.6 (7.6)	8.4 (7.7)	0.39*
Anger (mad, hate, kill etc.)	1.4 (3.9)	2.0 (3.1)	0.14
Number of reposts	0.7 (5.3)	19.3 (738.6)	0.04
Gender	male:247; female: 730	male:2682; female: 6090	/
Gender ratio	1.36	0.96	

*Indicates SMD > 0.2. Gender ratio for pro-cyberbullying posts = (Female user number support cyberbullying/Total number of female users)/(Male user number support cyberbullying/Total number of male users).

The number of pro-cyberbullying posts was around 10% of the number of anti-cyberbullying posts (see Table 5). Among the pro-cyberbullying posts, the average number of emotion words (affect) were significantly smaller than in anti-cyberbullying posts. Additionally, the average number of reposts in anti-cyberbullying posts was slightly larger than that in pro-cyberbullying posts (although the differences are not significant) according to the SMD threshold (i.e., SMD 0.2). The proportion of pro-cyberbullying posts from females is 1.36 times the number of posts from males, and the proportion of anti-cyberbullying posts from females is 0.96 times the number of posts from males.

### 4.2 Association of verification status and gender with emotion expressions in the pro-cyberbullying posts and anti-cyberbullying posts

We analyzed the differences of overall emotions (affect) and anger within pro-and anti-cyberbullying posts, the data analysis results are summarized in Table 3.

Table 3 shows that unverified male users used significantly more anger words than verified male users in pro-cyberbullying posts (SMD > 0.2 and the result is robust), while unverified male users have more overall emotion and anger expression than verified male users in anti-cyberbullying posts. Verified female users express significantly more anger (but not overall emotion) than verified male users in pro-cyberbullying posts (SMD > 0.2), while they express significantly more overall emotion than verified male users in anti-cyberbullying posts (SMD > 0.2). No significant difference shows in overall emotion and anger expression between verified female users and unverified female users (SMD < 0.2). These imply that unverified male users and verified female users were more likely to share pro-cyberbullying posts during the COVID-19 pandemic.

### 4.3 Differences in the reposts number of anti-cyberbullying and pro-cyberbullying posts

Table 4 summarizes pair-wise comparisons among several groups: verified male users as Type (1), unverified male users as Type (2), verified female users as Type (3), and unverified females as Type (4).

Table 4 shows that the number of reposts from verified male users was significantly larger than that from unverified male users and verified female users in pro-cyberbullying posts (SMD > 0.2). However, no significant differences were evident in the number of reposts between each pair of user groups in anti-cyberbullying posts (SMD < 0.2).

## 5 Discussion

### 5.1 Findings and practical implications

This study examined the pro-cyberbullying and anti-cyberbullying information sharing behavior of social media users regarding COVID-19 patients. We found that while there were a lot of posts about cyberbullying, the posts that could be considered pro-cyberbullying were in the minority (one in ten). Though only a small proportion of posts were pro-cyberbullying, this study suggests that the harmfulness of cyberbullying could be amplified, reflected in the large number of anger words in pro-cyberbullying posts from female verified users than the pro-cyberbullying posts from male verified users, as well as the larger number of female users in Weibo (Sheng et al., 2022). Practically, more attention should be paid to cyberbullying posts from verified male users because their posts had a significantly larger number of reposts than those from other users including male unverified users, and female users (verified or unverified).

Further, among all the pro-cyberbullying posts (977), 787 (80.5%) posts were from unverified users and 190 (19.5%) posts were from verified users. There are 4.14 times of pro-cyberbullying posts of the unverified users than that of the verified users. This study also found that users varied in expressing general emotions (affect) or anger in both pro-cyberbullying and anti-cyberbullying posts in accordance with their gender and verification status. Compared to unverified male users, verified male users have higher public visibility (verified users are more likely to be public figures) and may be more careful about maintaining a positive image. Expressing anger towards vulnerable groups is more likely to damage the image of verified users (Wang and Zhu, 2021).

Finally, expressing anger may be reasonable and could benefit people’s image in some circumstances. For instance, scapegoating strategy suggests that people/organizations might seek to shift blame to others (Coombs, 2015). Specifically, if individuals observe a negative outcome of others’ behaviors and attribute responsibility to him/her, they may feel anger and have negative feelings (Schwarz, 2012). In the anti-cyberbullying posts, female users (especially verified female users) expressed more emotions than male users (both verified and unverified), following social norms for emotional expression (Simon and Nath, 2004).

### 5.2 Theoretical implications

We found gender differences in sharing pro-cyberbullying posts of COVID-19 patients. The female to male ratio of 1.19 to 1 for pro-cyberbullying posts is slightly larger than the ratio of 0.93 for posts that are anti-cyberbullying. These findings may indicate that males are more cautious in sharing pro-cyberbullying posts than females. This discovery diverges from previous research, which, guided by self-control theory, suggested that females might be less inclined to engage in cyberbullying. However, it becomes reasonable when considering the perspective of the cyberbullying perpetrator, who deems others’ behavior as immoral. During the manual validation of pro-cyberbullying posts, it was evident that individuals who are pro-cyberbullying felt angered by what they perceived as the immoral actions of confirmed COVID-19 cases who ventured outdoors without wearing masks, thus endangering others. This phenomenon may be elucidated by Bandura’s moral agency theory (Kurtines and Gewirtz, 1991), which introduces the concept of moral disengagement—a cognitive process allowing individuals to act in a manner contrary to their personal and societal norms (Kurtines and Gewirtz, 1991). Therefore, we argue that in characterizing cyberbullying behavior through the lens of self-control theory, integrating moral agency theory is imperative.

Larger portion of pro-cyberbullying posts originated from unverified users rather than verified ones, lending support to the BCGM hypothesis, which proposes that perceived anonymity correlates positively with positive attitudes toward cyberbullying (Barlett et al., 2017). This discovery underscores the validity of using verification status to gauge the perceived anonymity of social media users. In addition, in the pro-cyberbullying posts, female users expressed more anger than male users (both verified and unverified). This finding suggests the potential of further considering gender as a moderating factor in the GST theory. In addition, compared with the verified male users, the unverified male users expressed more anger in pro-cyberbullying posts. This is reasonable, because the emotional responses of individuals are highly related to individuals’ cognitive processes (Armenti and Babcock, 2021), and people are cognitively inspired once they express anger at injustice and unethical phenomena. Verified females contradicting social norms and expressing more anger than verified males could also be understood through the lens of moral agency theory. This theory posits that individuals may justify actions that defy both their personal and societal norms, thus explaining the observed behavior (Kurtines and Gewirtz, 1991).

In summary, this study offers a new way to validate current theories on cyberbullying using social media data. By extracting the gender of social media users, we were able to test the self-control theory by analyzing the sharing behavior of males and females regarding pro-cyberbullying posts. Additionally, by examining the anonymity of social media users through their verification status, this study provides a new perspective for validating the BGCM. Through analyzing the ratio of anger/affect words in pro-cyberbullying posts among male/female users and verified/unverified users, this research has the potential to contribute to the development of new theories related to cyberbullying on social media.

### 5.3 Limitations and future research

However, this study had a number of limitations. Firstly, our research is based on Weibo data. Due to limitations with its open Application Programming Interface, we were unable to obtain a complete set of relevant data. Instead, we could only access a portion of randomly selected data and the sample size of pro-cyberbullying posts was relatively small compare with anti-cyberbullying posts. This implies that our research findings need further validation in larger datasets. Secondly, the keywords we choose are only representative keywords, but not a complete set of keywords which needs further studies to fill this gap. Thirdly, when categorizing the data related to cyberbullying, pro-cyberbullying, and anti-cyberbullying, we employed supervised machine learning techniques. However, the size of our testing set was small (e.g., in the second-round classification, the test set size is around 100, which may lead to the number of pro-cyberbullying posts is around 20), which suggests that there is need to enlarge of the dataset we now applied in the future. Fourthly, although efforts were made to ensure accurate translation, potential limitations inherent in the translation process from Chinese to English, such as cultural references and idiomatic expressions that may not fully convey the original intent or meaning. Lastly, the annotation set we used for identifying posts pro-or anti-cyberbullying was not extensive. Therefore, it is necessary for further research to expand such datasets and conduct research on them.

## 6 Conclusion

The rapid expansion of social media platforms over the past decade increased the prevalence of cyberbullying victimization and perpetration. Conducting investigations into cyberbullying is of great significance. The findings of this study provide gender-, verification status-, and emotion-specific empirical evidence about attitudes to cyberbullying in social media during a major public health crisis, which could be used to improve cyberbullying-post-identification algorithms. The study insights could also be employed by government agencies to mitigate the negative effects of online cyberbullying behaviors.

## Data availability statement

The raw data supporting the conclusions of this article will be made available by the authors, without undue reservation.

## Author contributions

LL: Conceptualization, Funding acquisition, Investigation, Methodology, Software, Visualization, Writing – original draft, Writing – review & editing. JZ: Methodology, Validation, Writing – review & editing. SM: Supervision, Validation, Writing – review & editing, Funding acquisition. RS: Supervision, Validation, Writing – review & editing, Funding acquisition. AR: Funding acquisition, Supervision, Validation, Writing – review & editing.

## Footnotes

^1^ https://index.baidu.com/v2/index.html

^2^ https://trends.google.com/trends/

^3^Chinese keywords are as follows: “毒王(不省心), 千里投毒 (确诊者不省心), 密接不省心,确诊者乱跑, 密接者乱跑, 毒王乱跑, 毒王烦人,确诊者烦人, 密接烦人,毒王要死了,确诊者要死了,密接要死了.”

^4^ https://open.weibo.com/

^5^ https://pypi.org/project/jieba/

^6^ https://github.com/sunshinegirl5566/pro-cyberbullying-data/tree/main
